# Supplementary material for: EnzML: multi-label prediction of enzyme classes using InterPro signatures
Source: BMC Bioinformatics. 2012 Apr 25;13:61. doi: 10.1186/1471-2105-13-61 (PMC3483700; doi:10.1186/1471-2105-13-61)
Supplement: Addtional file 5 — The Java code to format the data files, evaluate and predict. The file enzml_java_code.tar.gz contains the Java code used to format database data to ARFF and XML formats, to execute cross and train-test (jackknife) evaluations and to record evaluation results to database. More information is included in the readme.txt file and the Javadoc files. The code can be used with a MySQL database. To use a different database software, other JDBC drivers might be required. [file 1471-2105-13-61-S5.gz › java_code/enzml2011/doc/uk/ac/ed/inf/enzml/weka/DataSetWriter.html]

DataSetWriter


---


|  |  |  |
| --- | --- | --- |
| **Overview** \| **Package** \| **Class** \| **Use** \| **Tree** \| **Deprecated** \| **Index** \| **Help** | |  |
| **PREV CLASS**   **NEXT CLASS** | **FRAMES**    **NO FRAMES**     **All Classes** |  |
| SUMMARY: NESTED \| FIELD \| CONSTR \| METHOD | DETAIL: FIELD \| CONSTR \| METHOD |  |


---


## uk.ac.ed.inf.enzml.weka Class DataSetWriter

```
java.lang.Object
  uk.ac.ed.inf.enzml.weka.DataSetWriter
```

---

``` public class DataSetWriter extends java.lang.Object ```

Class

**Version:**
:   3 Sep 2010

**Author:**
:   Luna De Ferrari luna.deferrari-at-ed.ac.uk

---

**Constructor Summary**

`DataSetWriter(DataSetManager manager)`


| **Method Summary** | |
| --- | --- |
| `java.lang.String` | `attributesSection(weka.core.Instances instances)` |
| `java.lang.String` | `dataStatistics()` |
| `DataSetManager` | `getDataSetManager()` |
| `java.lang.String` | `getFilePath()` |
| `java.lang.String` | `getLog()` |
| `java.lang.String` | `logHeader()` |
| `java.lang.String` | `logTail()` |
| `void` | `writeAttributesAndClasses(weka.core.Instances instances)`             Write the data instances to file |
| `void` | `writeDataStatistics()`             Logs to file the number of instances, classes and attributes in the dataset. |
| `void` | `writeHeader()` |
| `void` | `writeInstanceToFile(java.lang.String instanceName, weka.core.Instance instance)`             Write an individual Weka instance to file |
| `void` | `writeTail()` |

**Methods inherited from class java.lang.Object**

`equals, getClass, hashCode, notify, notifyAll, toString, wait, wait, wait`

**Constructor Detail**

### DataSetWriter

```
public DataSetWriter(DataSetManager manager)
```


**Method Detail**

### attributesSection

```
public java.lang.String attributesSection(weka.core.Instances instances)
```

---


### dataStatistics

```
public java.lang.String dataStatistics()
```

---


### getDataSetManager

```
public DataSetManager getDataSetManager()
```

---


### getFilePath

```
public java.lang.String getFilePath()
```

---


### getLog

```
public java.lang.String getLog()
```

---


### logHeader

```
public java.lang.String logHeader()
```

---


### logTail

```
public java.lang.String logTail()
```

---


### writeAttributesAndClasses

```
public void writeAttributesAndClasses(weka.core.Instances instances)
```

:   Write the data instances to file

---


### writeDataStatistics

```
public void writeDataStatistics()
```

:   Logs to file the number of instances, classes and attributes in the
    dataset.

---


### writeHeader

```
public void writeHeader()
```

---


### writeInstanceToFile

```
public void writeInstanceToFile(java.lang.String instanceName,
                                weka.core.Instance instance)
```

:   Write an individual Weka instance to file

---


### writeTail

```
public void writeTail()
```


---


|  |  |  |
| --- | --- | --- |
| **Overview** \| **Package** \| **Class** \| **Use** \| **Tree** \| **Deprecated** \| **Index** \| **Help** | |  |
| **PREV CLASS**   **NEXT CLASS** | **FRAMES**    **NO FRAMES**     **All Classes** |  |
| SUMMARY: NESTED \| FIELD \| CONSTR \| METHOD | DETAIL: FIELD \| CONSTR \| METHOD |  |


---
